# Effect of Sintering Temperature on the Microstructure and Mechanical Properties of Multidoped Zirconia-Toughened Alumina

**DOI:** 10.3390/ma19214436

**Published:** 2026-10-22

**Authors:** Yuriy Garanin, Malik Kaliyekperov, Dias Alkenov, Rafael Shakirzyanov

**Affiliations:** 1 Astana Branch of the Institute of Nuclear Physics, Astana 010000, Kazakhstan; kaliekperov_mye_2@enu.kz (M.K.); d.alkenov@icloud.com (D.A.); shakirzyanov_ri@enu.kz (R.S.); 2 Engineering Laboratory, L.N. Gumilyov Eurasian National University, Astana 010000, Kazakhstan

**Keywords:** ZTA, multidoped zirconia, microstructure, sintering temperature, mechanical properties, fracture toughness

## Abstract

**Highlights:**

- ZTA ceramics containing Zr_0.90_(Y, Ce, Ca, Mg)_0.10_O_2_ were sintered at 1400–1600 °C.
- The phase composition remained largely unchanged with sintering temperature.
- Higher sintering temperatures promoted alumina and zirconia grain growth.
- The ceramics achieved a flexural strength of 620 MPa.

**Abstract:**

In this study, the effect of sintering temperature on the phase composition, density, microstructure, and mechanical properties of multidoped zirconia-toughened alumina (MZTA) ceramics containing multidoped zirconia (Zr_0.90_(Y, Ce, Ca, Mg)_0.10_O_2_) was investigated. The samples were fabricated using a conventional ceramic processing and sintered at temperatures ranging from 1400 to 1600 °C for 5 h. X-ray diffraction and whole-powder-pattern fitting (WPPF) analysis showed that, regardless of the sintering temperature, the samples consisted predominantly of t-ZrO_2_ and Al_2_O_3_ phases, with a minor (0.7–2.8 wt. %) amount of m-ZrO_2_ detected at certain temperatures. Scanning electron microscopy revealed that increasing the sintering temperature from 1400 to 1600 °C resulted in an increase in the average Al_2_O_3_ grain size from 0.39 to 1.32 µm and the ZrO_2_ grain size from 0.28 to 0.92 µm. The sample sintered at 1500 °C exhibited the best combination of microhardness (HV5 1748) and flexural strength (620 MPa), owing to its high density and homogeneous microstructure. At higher sintering temperatures, abnormal grain growth accompanied by a decrease in density was observed, resulting in a reduction in flexural strength. The fracture toughness (K_IC_) showed an approximately linear increase with increasing sintering temperature.

## 1. Introduction

Zirconia toughened alumina (ZTA) is widely used in the manufacture of cutting tools, dies, armor, biomedical implants, and prosthetic components [1,2,3,4,5]. Despite its high hardness and compressive strength, Al_2_O_3_ has several limitations associated with its low fracture toughness and flexural strength [6,7,8]. The incorporation of ZrO_2_ into the Al_2_O_3_ matrix can significantly improve fracture toughness and flexural strength through several mechanisms, including transformation toughening associated with the stress-induced transformation of metastable tetragonal ZrO_2_ into the monoclinic phase (t → m transformation), stresses arising from the difference in the coefficients of thermal expansion of Al_2_O_3_ and ZrO_2_, and the suppression of abnormal grain growth through the pinning effect [9,10]. The final properties of ZTA depend on several factors, including the type of ZrO_2_ stabilizer and the presence of various additives, the ZrO_2_ content in the Al_2_O_3_ matrix, and the sintering temperature and holding time [11,12,13].

ZrO_2_ is a polymorphic material that exhibits three crystal structures depending on temperature: monoclinic (m-ZrO_2_) from room temperature to 1170 °C, tetragonal (t-ZrO_2_) from 1170 to 2379 °C, and cubic (c-ZrO_2_) above 2370 °C [14]. The phase transformation between the monoclinic and tetragonal phases is martensitic in nature and is accompanied by a volume change of approximately 4.5%, which, under uncontrolled conditions, can lead to cracking and complete failure of the material during sintering [15].

High-temperature t- and c- ZrO_2_ phases can be stabilized by doping with other metal oxides [16,17,18], tailoring the grain size [19,20] and residual stress control [21]. The most widely used approach to stabilization is doping with compounds containing cations that differ from Zr^4+^ in valence (e.g., Y_2_O_3_, MgO, and CaCO_3_) and/or ionic radius (e.g., CeO_2_, Ta_2_O_5_, etc.) [22,23,24]. This approach enables (1) the generation of oxygen vacancies to maintain charge neutrality [25] and (2) the development of significant compressive or tensile stresses within the crystal lattice [26,27]. Both factors play a key role in stabilizing the high-temperature ZrO_2_ phases at room temperature. However, the use of a single dopant for ZrO_2_ stabilization has several limitations. Despite their high flexural strength (~1200 MPa) and fracture toughness (6–9 MPa∙m^1/2^), Y_2_O_3_-doped ceramics are susceptible to low-temperature degradation (LTD), which involves the spontaneous t → m phase transformation in humid environments [28,29]. LTD results in increased surface roughness, grain pull-out, crack formation, and degradation of the mechanical properties [30]. In contrast, CeO_2_-doped ZrO_2_ ceramics are resistant to LTD but exhibit significantly lower hardness (9–15 GPa) and flexural strength (300–600 MPa) [31]. The use of CaO and MgO as dopants is limited by eutectoid decomposition, which leads to the degradation of mechanical properties during thermal cycling above ~1200 °C [24]. To combine the advantages of different dopants while mitigating their limitations, multi-element doping has been proposed. Co-doping with CaCO_3_ and Y_2_O_3_ to stabilize the t-ZrO_2_ phase significantly improves resistance to LTD [32], while the addition of CeO_2_ to MgO-doped ZrO_2_ enhances thermal stability and compressive strength [33]. Our previous study has shown that multidoping ZrO_2_ with yttrium, cerium, magnesium, and calcium oxides has the potential to suppress hydrothermal aging and improve its mechanical properties [34]. However, their application is limited by excessive sintering activity, which makes it difficult to achieve optimal density and microstructural homogeneity in bulk samples. In contrast, the more rigid Al_2_O_3_ matrix is known to constrain abnormal ZrO_2_ grain growth and generate significant mechanical stresses, which provide an additional mechanism for stabilizing the t-ZrO_2_ phase [35].

Several studies have shown that co-doping with multiple t-ZrO_2_ stabilizers can significantly improve the mechanical properties of ZTA ceramics. The incorporation of various combinations of oxide additives (Y_2_O_3_, CeO_2_, MgO, CaCO_3_, Cr_2_O_3_, etc.) into ZTA enables its mechanical behavior to be tailored through modifications of the phase composition, grain size, and grain morphology. The combined addition of Y_2_O_3_ and CeO_2_ suppresses Al_2_O_3_ grain growth and improves hardness and flexural strength through grain-growth inhibition and the formation of secondary phases [36]. The MgO-CeO_2_-ZTA system demonstrates excellent fracture toughness (K_IC_ = 12.1 MPa∙m^1/2^), hardness (1784 HV), and flexural strength (703 MPa), which are attributed to the formation of elongated MgAl_11_CeO_19_ grains and effective grain-growth control. The elongated grains promote crack deflection along the grain boundaries, thereby increasing the crack propagation path and the energy required for crack propagation [37].

The ZrO_2_ content in the Al_2_O_3_ matrix significantly affects the mechanical properties and grain growth behavior of ZTA ceramics. At least 7 wt.% ZrO_2_ is required to suppress abnormal grain growth, which has been attributed to the need for ZrO_2_ particles to surround all four faces of the Al_2_O_3_ grains [38]. However, an optimal combination of high hardness, fracture toughness, and flexural strength can be achieved at significantly higher ZrO_2_ contents. For example, Ganesh et al. showed that the addition of 20 wt.% stabilized ZrO_2_ increased the fracture toughness from 2.5 to 4.6 MPa∙m^1/2^ and the flexural strength from 150 to 400 MPa compared with Al_2_O_3_ [39]. In another study, Li et al. reported that the highest flexural strength (694 MPa) among the investigated compositions was also achieved with the addition of 20 wt.% ZrO_2_ [40]. Increasing the ZrO_2_ content above 20 wt.% is generally not considered, as it does not provide a significant improvement in fracture toughness or flexural strength but increases the risk of spontaneous t → m phase transformation in humid environments [41].

The effect of sintering temperature on the phase composition, microstructure, and mechanical properties of ZTA ceramics has been extensively studied for conventional composites containing ZrO_2_ stabilized with one or two oxides [39,42,43]. It has been shown that increasing the sintering temperature promotes densification and reduces residual porosity. However, it may also lead to abnormal grain growth, pore entrapment, and ZrO_2_ agglomeration [44,45]. Fan et al. investigated the effect of sintering temperature on MgO- and TiO_2_-doped ZTA ceramics over the temperature range of 1100–1400 °C [45]. Despite the progressive increase in the m-ZrO_2_ fraction at higher sintering temperatures, the sample sintered at 1450 °C exhibited the most favorable combination of mechanical properties. Al-Amin et al. examined the effect of sintering temperature on ZTA ceramics doped with MgO, TiO_2_, and CeO_2_ over the temperature range of 1450–1650 °C [46]. A sintering temperature of 1500 °C was identified as optimal, yielding a relative density of 94.8%, a fracture toughness (K_IC_) of 12 MPa∙m^1/2^, and a hardness of 14.2 GPa. A further increase in sintering temperature promotes abnormal grain growth, resulting in pore entrapment at grain boundaries and triple junctions and, consequently, deterioration of the mechanical properties. Sintering temperature is therefore a critical processing parameter that strongly influences the final properties of ZTA ceramics. However, little information is available on the effect of sintering temperature on the phase composition, sintering behavior, and mechanical properties of ZTA ceramics containing ZrO_2_ co-doped with yttrium, cerium, calcium, and magnesium oxides.

In this study, the effect of sintering temperature in the range of 1400–1600 °C on the phase composition, surface morphology, and mechanical properties of multidoped zirconia-toughened alumina (MZTA) ceramics was investigated. Particular attention was paid to the relationship between sintering temperature, surface morphology, and mechanical properties.

## 2. Materials and Methods

### 2.1. Synthesis of α-Al_2_O_3_ Particles

α-Al_2_O_3_ particles were synthesized by a co-precipitation method using a 0.1 mol/L solution of Al(NO_3_)_3_∙9H_2_O (Sigma-Aldrich, St. Louis, MO, USA) prepared in a distilled water. A 25% NH_3_∙H_2_O (Sigma-Aldrich, St. Louis, MO, USA) solution was added as the precipitating agent until a pH of 9 was reached. The precipitate was separated by repeated centrifugation at 5000 rpm for 5 min. The resulting precipitate was then dried at 45 °C for 24 h.

Crystallization of the obtained precipitate was performed in two stages. First, the particles were calcined in a Nabertherm LHT 08/18 muffle furnace (Nabertherm GmbH, Lilienthal, Germany) at 700 °C for 30 min. The calcined powder was subsequently milled in a Pulverisette 7 premium line planetary ball mill (Fritsch, Idar-Oberstein, Germany) at 300 rpm for 3 h to break up large agglomerates. In the second stage, the resulting powder was heat-treated in a muffle furnace at 1200 °C for 30 min to promote the formation of the α-Al_2_O_3_ phase.

### 2.2. Synthesis of Zr_0.90_(Y, Ce, Ca, Mg)_0.10_O_2_ Nanoparticles

Zr_0.90_(Y, Ce, Ca, Mg)_0.10_O_2_ nanoparticles were synthesized by a low-temperature hydrothermal method at 130 °C with a holding time of 12 h. ZrOCl_2_·8H_2_O, Ce(NO_3_)_3_·6H_2_O, Y(NO_3_)_3_·6H_2_O, Mg(NO_3_)_2_·6H_2_O, and CaCl_2_ (all Sigma-Aldrich, St. Louis, MO, USA) were used as precursors and dissolved in distilled water to obtain a solution with a total precursor concentration of 0.1 mol/L. The Y:Ce:Ca molar ratio in the solution was 1:1:1:1. A 1 mol/L KOH (Sigma-Aldrich, St. Louis, MO, USA) solution prepared in distilled water was used as a mineralizer and added until a pH of 9 was reached. Following hydrothermal synthesis, the resulting precipitate was washed by repeated centrifugation at 5000 rpm for 5 min and subsequently dried at 45 °C for 24 h. The dried powder was then calcined in a Nabertherm LHT 08/18 muffle furnace (Nabertherm GmbH, Lilienthal, Germany) at 650 °C for 30 min to promote crystallization and remove residual synthesis products, including physically and chemically adsorbed water and –OH groups.

### 2.3. Preparation of MZTA Ceramic Samples

To prepare the MZTA samples, the pretreated α-Al_2_O_3_ and Zr_0.90_(Y, Ce, Ca, Mg)_0.10_O_2_ powders were mixed at a weight ratio of 80:20, respectively. The powders were homogenized in a Pulverisette 7 premium line planetary ball mill (Fritsch, Idar-Oberstein, Germany) using a ZrO_2_ milling bowl and balls at 500 rpm for 10 h. Wet milling was performed with the addition of isopropanol to improve homogenization and promote further particle size reduction. After milling, the mixture was dried at 60 °C for 12 h and subsequently passed through a 45 µm sieve to remove possible large agglomerates. Polyvinyl alcohol (PVA) was added to the resulting powder mixture at a concentration of 5 wt.% as a plasticizer. The prepared powder was uniaxially pressed in a steel die at 450 MPa using a PARATUSpress P115 hydraulic press (Paratus, Chelyabinsk, Russia). The resulting green bodies were subjected to a two-stage heat treatment in a Nabertherm LHT 08/18 muffle furnace (Nabertherm GmbH, Lilienthal, Germany). In the first stage, debinding was carried out at 500 °C for 30 min using a heating rate of 0.5 °C/min to remove PVA. In the second stage, the resulting brown bodies were sintered at temperatures ranging from 1400 to 1600 °C in 50 °C increments for 5 h to determine the optimal sintering temperature.

### 2.4. Sample Characterization

X-ray diffraction (XRD) was performed using a Rigaku SmartLab diffractometer (Rigaku, Tokyo, Japan) with CuKα radiation in Bragg–Brentano geometry. The PDF-2 (Powder Diffraction File) database was used for phase identification. Quantitative phase and structural analyses were carried out by whole-powder-pattern fitting (WPPF) using the Powder XRD Plugin implemented in the Rigaku Smartlab software (Rigaku, Tokyo, Japan, version v4.6.671.0) [47,48]. Surface morphology was examined by scanning electron microscopy (SEM) using a Phenom ProX G6 scanning electron microscope (Thermo Fisher Scientific, Eindhoven, The Netherlands) equipped with an energy-dispersive X-ray spectroscopy (EDX) system for elemental analysis. The average grain size was determined from the SEM images using ImageJ software (version v1.54t, National Institutes of Health, Bethesda, MD, USA). 

Surface morphology and elemental composition were examined by scanning electron microscopy (SEM) using a Phenom ProX G6 scanning electron microscope (Thermo Fisher Scientific, Eindhoven, The Netherlands) equipped with an energy-dispersive X-ray spectroscopy (EDX) system. The average grain size was calculated using ImageJ software. 

The geometrical density was determined as the ratio of the sample mass to its volume:

(1) $$\rho =\frac{m}{V}$$

where m is the sample mass and V is the volume calculated from the geometrical dimensions of the sample.

The theoretical density of the MZTA specimen was calculated using the rule of mixtures:

(2) $$\rho_{\mathrm{th}}={\left(\sum w_{i}/\rho_{i}\right)}^{-1}$$

where w_i_ and ρ_i_ are the mass fraction and theoretical density of the corresponding component, respectively.

Vickers microhardness was measured using DuroScan-50 microhardness tester (EMCO-TEST Prüfmaschinen GmbH, Kuchl, Austria). Prior to testing, the sample surfaces were ground and polished using a Tegramin preparation system (Struers, Ballerup, Denmark). Measurements were performed at loads corresponding to HV1, HV2, HV5, HV10, and HV20. Five indentations were made at each load, and the mean values and corresponding measurement errors were calculated. The distance between the centers of adjacent indentations was maintained at 100–200 µm to minimize interactions between the associated plastic deformation zones.

The fracture toughness (K_IC_) of the samples was evaluated from the lengths of cracks generated during Vickers indentation. For each sintering temperature, 10 measurements were performed, and the standard deviation was calculated. Different analytical models were used to calculate K_IC_ depending on the relationship between the crack length and indentation size. When the ratio of the half-diagonal of the indentation (f) to the crack length (k) was ≤2.5 (f/k ≤ 2.5), the cracks were classified as Palmqvist-type cracks. In this case, K_IC_ was calculated using the following expression [49]:

(3) $$K_{\mathrm{IC}}=0.035{\left(\frac{E\phi }{\mathrm{HV}}\right)}^{2/5}\cdot (\frac{\mathrm{HV}\sqrt{f}}{\phi })\cdot {\left(\frac{k}{f}\right)}^{-1/2}$$

where E—Yong modulus (340 GPa); HV—microhardness; *ϕ*—constraint factor (for ceramics *ϕ* = 3); f—the indentation half-diagonal; and k—crack length.

When the f/k ratio was ≥2.5, the cracks were classified as radial-median cracks, and K_IC_ was calculated using the following expression [50]:

(4) $$K_{\mathrm{IC}}=0.016{\left(\frac{E}{\mathrm{HV}}\right)}^{1/2}\cdot {\left(\frac{k}{f}\right)}^{-1/2}$$

The strength of the samples was evaluated by determining the flexural strength using a piston-on-three-ball test [51]:

(5) $$\sigma_{\mathrm{flex}}=\frac{3\left(1+\nu \right)P}{4\pi t^{2}}\left[1+2\ln\frac{a}{b}+\frac{\left(1-\nu \right)}{\left(2+\nu \right)}\left(1-\frac{b^{2}}{2a^{2}}\right)\frac{a^{2}}{R^{2}}\right]$$

where σ_flex_—flexural strength; P—load in newtons (N); υ—Poisson’s ratio (0,28); t—disc thickness; a—radius of the support circle on which the spherical supports are positioned; b—radius of the loading piston in contact with the disc; and R—radius of the ceramic disc.

The load during compression testing was measured using a WalterBai LFM-L 10 kN universal electromechanical single-column testing machine (Walter + Bai AG, Löhningen, Switzerland). For each sintering temperature, five measurements were performed to determine the mean and standard deviation, ensuring the reliability of the results.

## 3. Results

**Figure 1:**
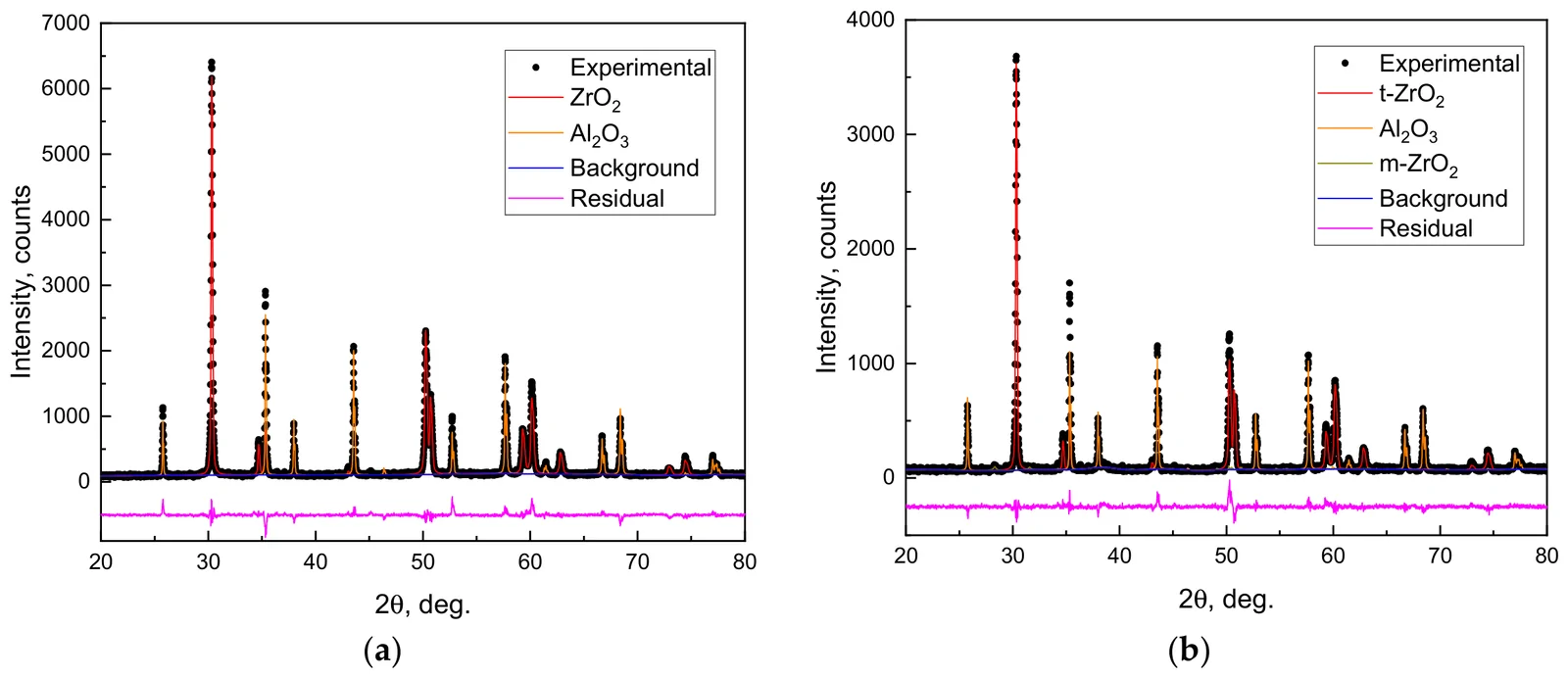

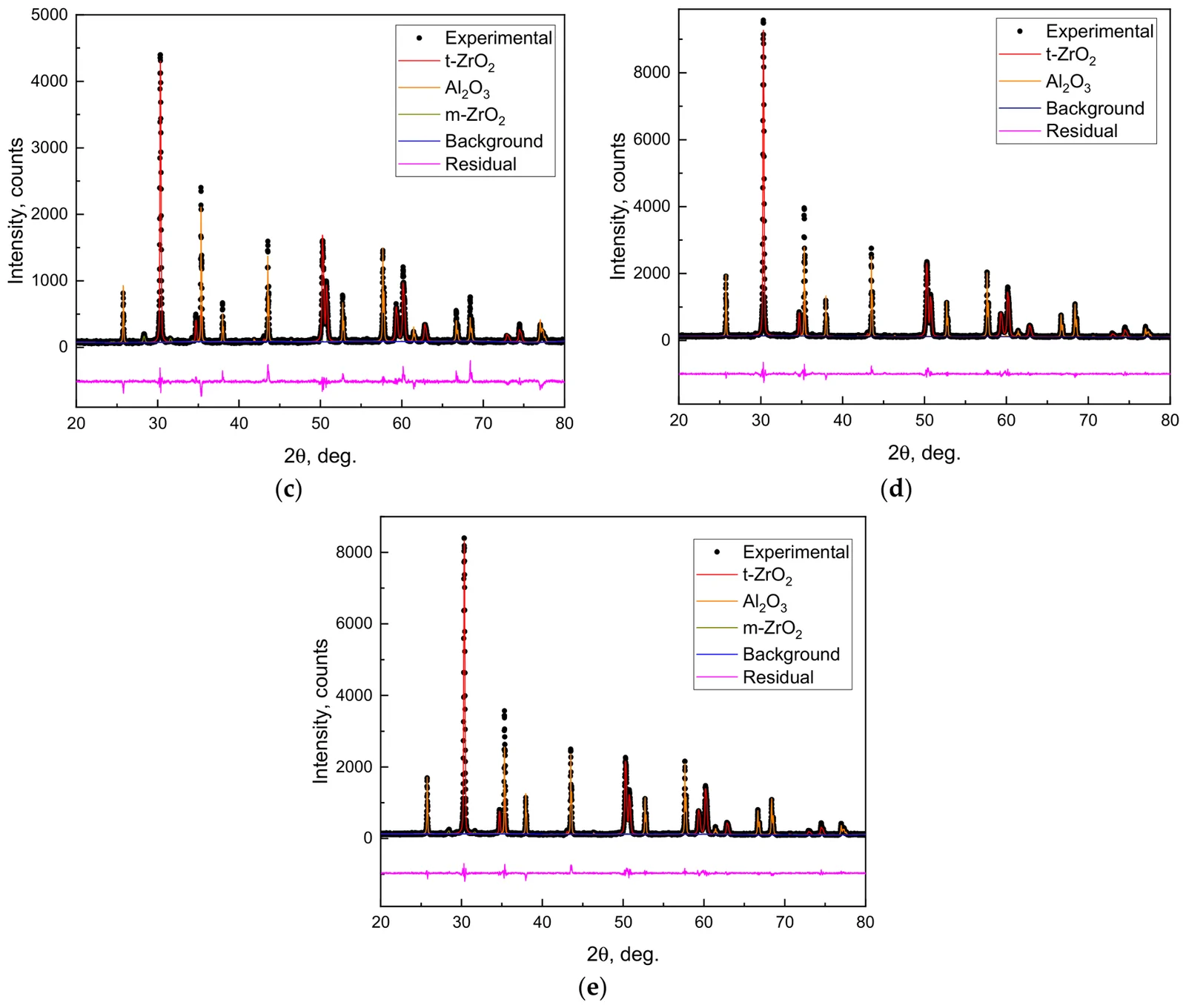
WPPF refinement of the phase composition of MZTA ceramics sintered at different temperatures: (**a**) 1400 °C, (**b**) 1450 °C, (**c**) 1500 °C, (**d**) 1550 °C, and (**e**) 1600 °C.

Figure 1 shows the XRD patterns of the MZTA ceramics sintered at different temperatures. Regardless of the sintering temperature, all samples contain α-Al_2_O_3_ and t-ZrO_2_ phases. A minor amount of the m-ZrO_2_ phase was detected in the samples sintered at 1450, 1500, and 1600 °C (Table 1). During powder processing, an increase in the zirconium content relative to the nominal composition was detected. This deviation is attributed to zirconia incorporation during the intensive milling stage. The formation of the monoclinic phase may be associated with partial destabilization of the tetragonal phase during high-temperature sintering and subsequent cooling. The stability of the t-ZrO_2_ phase can be attributed to a combination of factors, including the concentration and distribution of stabilizing cations and the grain size. An increase in sintering temperature may promote ZrO_2_ grain growth, thereby reducing the contribution of size-induced stabilization of the tetragonal phase and increasing its susceptibility to spontaneous t → m transformation [52]. 

Another contributing factor may be the redistribution of dopant cations between the grain interiors and grain-boundary/interphase regions during sintering, leading to local variations in the degree of t-ZrO_2_ stabilization [53]. Nevertheless, the fraction of m-ZrO_2_ remains low (0.7 wt. %) even after sintering at 1600 °C, while t-ZrO_2_ remains the predominant zirconia phase. This indicates the relatively high stability of the tetragonal phase in the MZTA composite. One possible reason for its stability is the constraint imposed on ZrO_2_ grain growth by the surrounding Al_2_O_3_ matrix, which suppresses the pronounced grain coarsening previously observed in single-phase multidoped zirconia ceramics [34].

**Figure 2:**
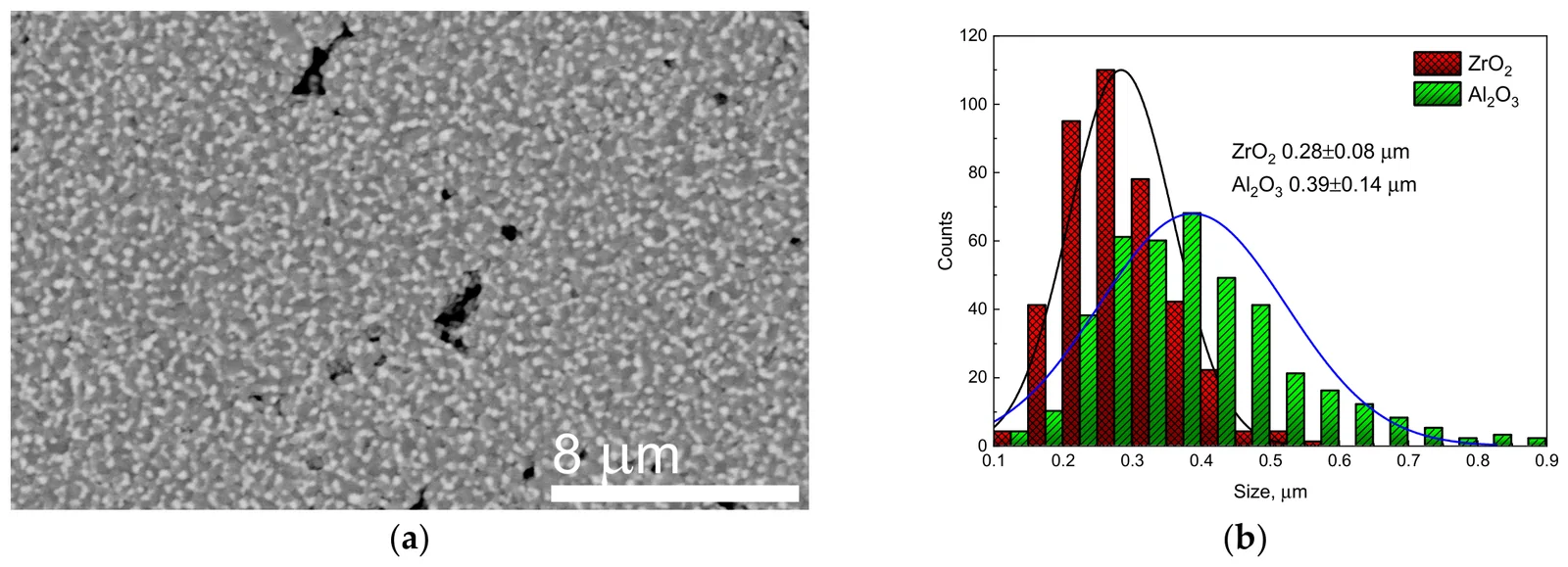

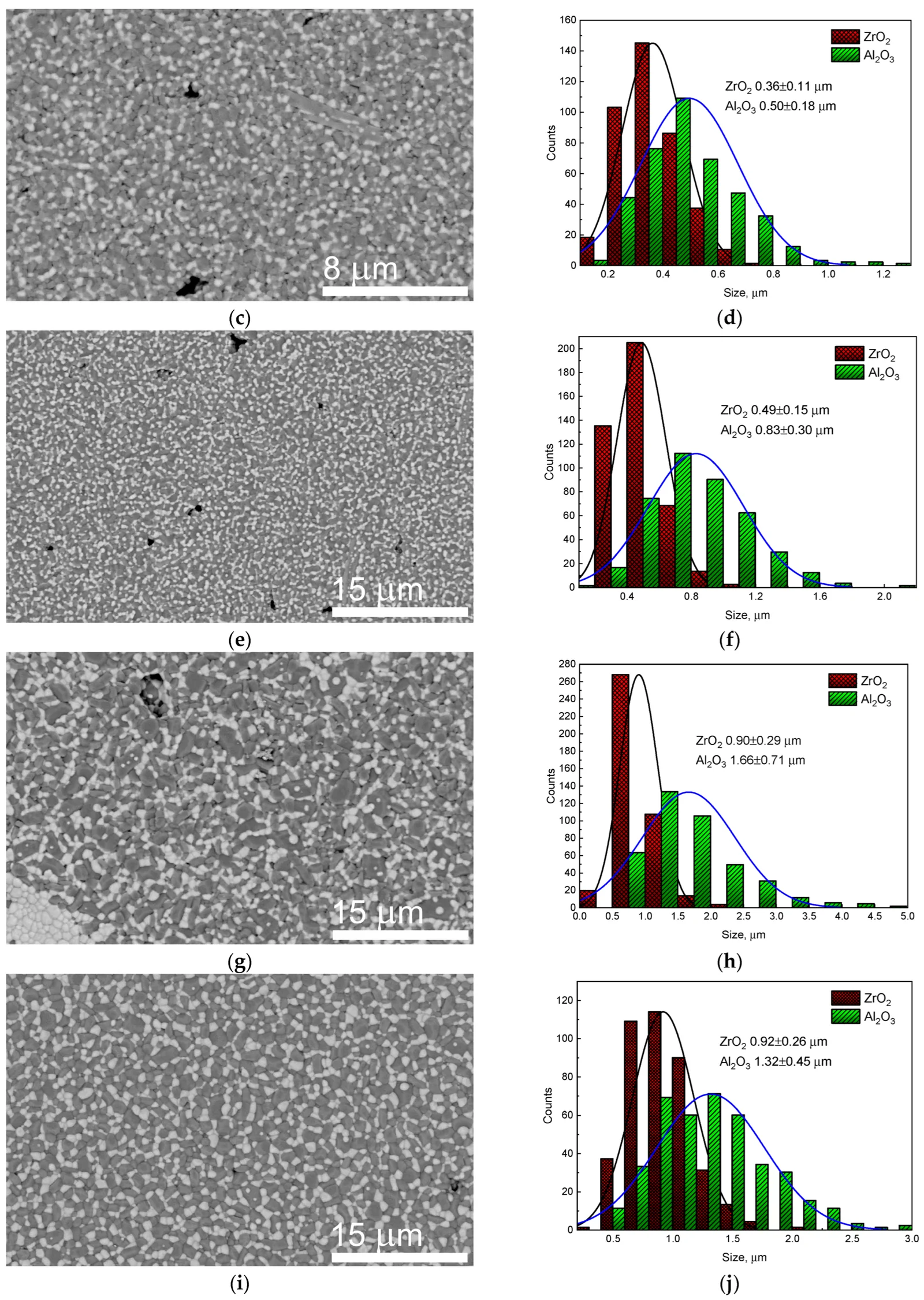
SEM images and corresponding grain size distributions of MZTA samples sintered at different temperatures (**a**,**b**) 1400 °C, (**c**,**d**) 1450 °C, (**e**,**f**) 1500 °C, (**g**,**h**) 1550 °C, and (**i**,**j**) 1600 °C, respectively.

SEM analysis of the surface morphology (Figure 2) revealed a pronounced evolution of the sample microstructure with increasing sintering temperature. As the temperature increased from 1400 to 1600 °C, the average size of the darker grains, identified as Al_2_O_3_, increased from 0.39 to 1.32 µm, while that of the brighter grains, assigned to ZrO_2_, increased from 0.28 to 0.92 µm. This progressive grain growth was accompanied by a reduction in the number of small surface pores and the development of a denser microstructure.

**Figure 3:**
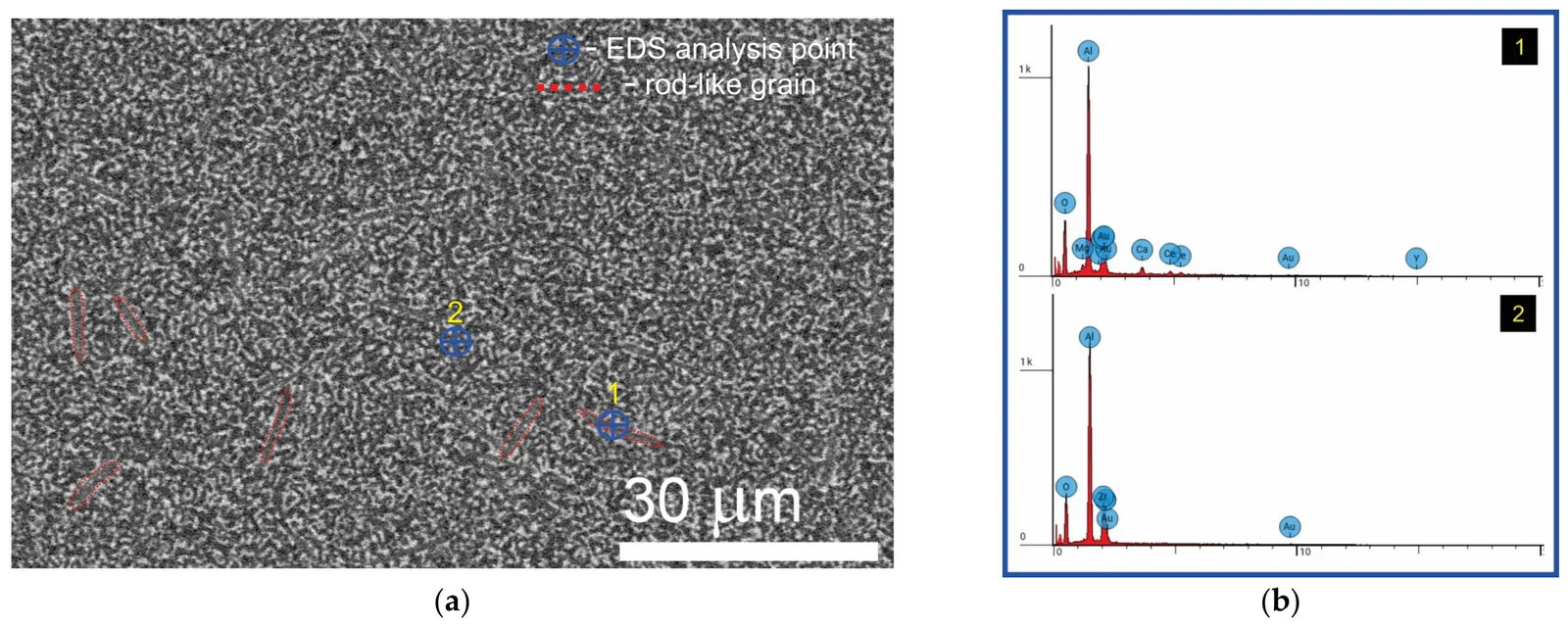
(**a**) SEM images of the surface of MZTA ceramics sintered at 1550 °C with the marked points for EDS analysis, and (**b**) corresponding EDS spectra of points 1 and 2.

A characteristic microstructural feature of all the investigated samples is the presence of isolated elongated, rod-like Al_2_O_3_ grains (Figure 3). One possible explanation for this behavior is the presence Ce, Mg, and Ca cations, which can modify the state and mobility of Al_2_O_3_ grain boundaries. Local redistribution of these elements during high-temperature sintering may induce differences in the mobility of individual grain boundaries, thereby promoting the preferential growth of a limited number of grains [54,55]. Local EDS analysis provides further evidence of chemical heterogeneity in these regions: the signals corresponding to Ce, Mg, and Ca are considerably stronger in the vicinity of the elongated Al_2_O_3_ grains that in the surrounding matrix. This observation suggests preferential redistribution of segregation of these elements in regions associated with abnormal Al_2_O_3_ grain growth. However, the spatial resolution of EDS is insufficient to determine unambiguously whether these elements are located within the Al_2_O_3_ grains, at grain or interphase boundaries, or in the adjacent regions. 

**Figure 4:**
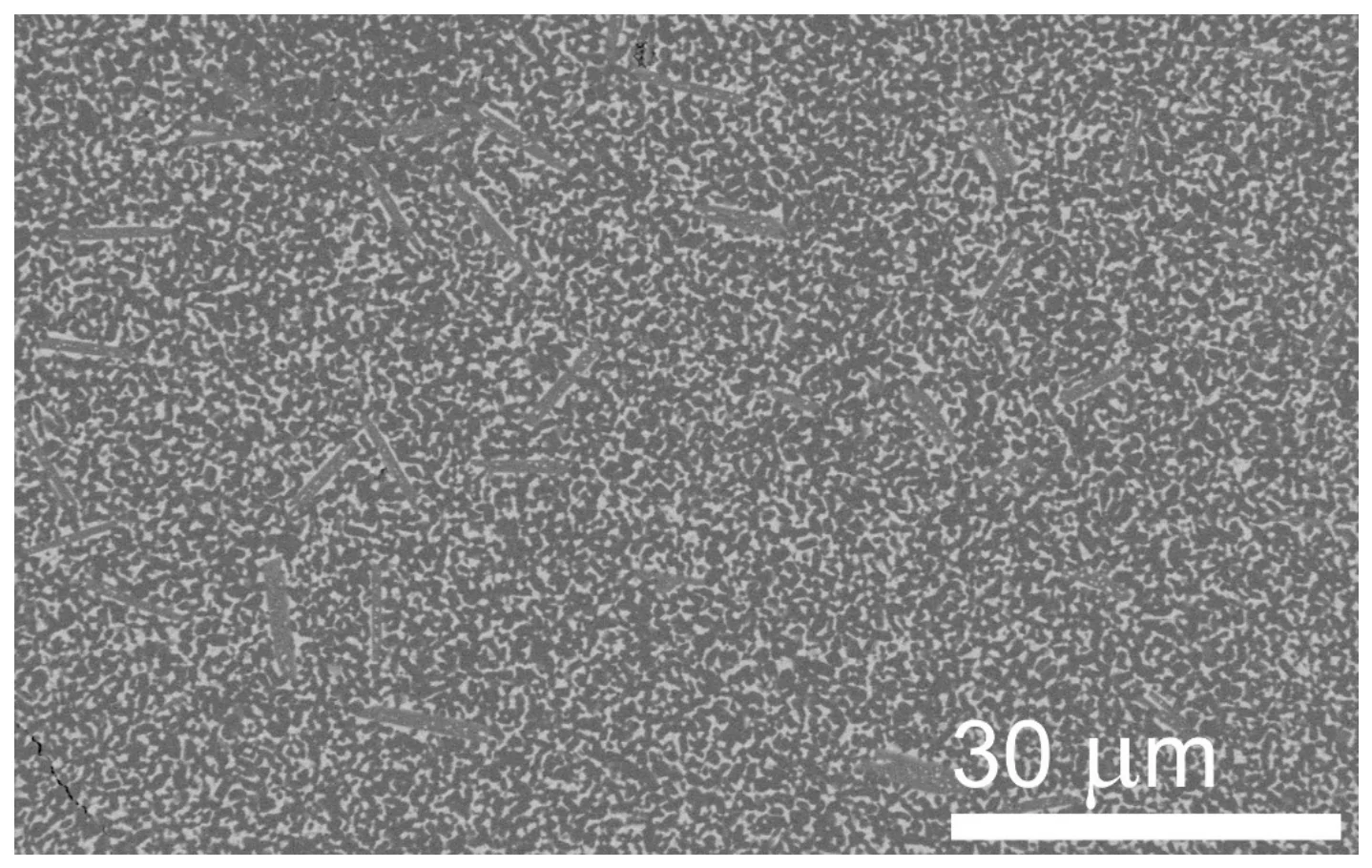
SEM image of the surface of the MZTA sample sintered at 1550 °C after polishing.

In addition to changes in the size of individual grains, localized regions containing agglomerated Zr_0.90_(Y, Ce, Mg, Ca)_0.10_O_2_, approximately 20–50 µm in size, were identified in the samples sintered at 1450 and 1550 °C. The presence of these regions indicates that a certain degree of microstructural heterogeneity persists after sintering. Their formation could initially be attributed to insufficient homogenization of the starting powder mixture. However, this explanation is inconsistent with the observations of the polished surfaces (Figure 4). After removal of the surface layer by polishing, such regions were no longer observed. This finding indicates that the detected chemical and microstructural heterogeneity is predominantly confined to near-surface region and suggests that insufficient homogenization of the powder mixture is unlikely to be its primary origin. The formation of ZrO_2_-rich regions may instead be associated with mass-transport processes and microstructural evolution at the free surface during high-temperature sintering.

**Figure 5:**
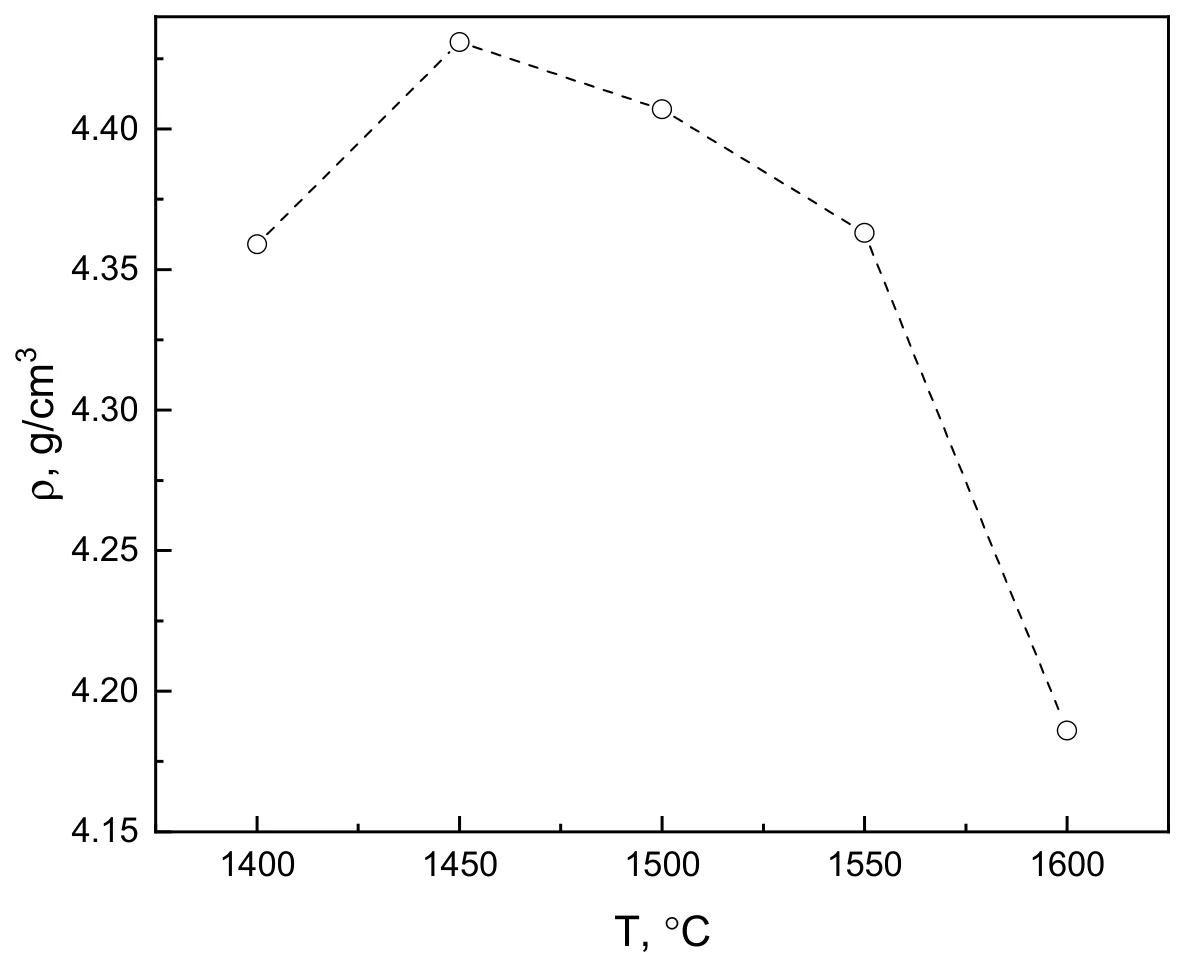
Density of MZTA samples as a function of sintering temperature.

Despite the apparent densification of the microstructure with increasing sintering temperature observed by SEM, the density measurements (Figure 5) show the opposite trend. This behavior can be explained by grain coarsening becoming dominant over densification at higher sintering temperatures (abnormal grain growth). Accelerated grain-boundary migration may cause the boundaries to detach from the pores, resulting in pore entrapment within the growing grains. Consequently, the residual porosity becomes predominantly closed and intragranular. Such pores may not be visible in SEM images of the surface but can still reduce the bulk density of the ceramics. 

Two mechanisms can be associated with abnormal or exaggerated grain growth. First is a small amount of the liquid phase, forming during sintering. At high temperature eutectoid decomposition of Zr_0.90_(Y, Ce, Ca, Mg)_0.10_O_2_ can occur, which leads to the formation of CaO grains in the volume of ceramics. In this case, tricalcium aluminate, Ca_3_Al_2_O_6_, with melting point 1542 °C can be formed [56]. With liquid phase formation (even when liquid quantity is very low), the growth rate of crystal rockets and leads to the formation of elongated crystals in the microstructure of alumina ceramics [57]. The second mechanism can be described as follows. It is known that the alumina crystal lattice (with space group R3c) can be described as a close-packed hexagonal arrangement of oxygen ions, where metal cations occupy interstitial sites. During sintering there is a high possibility of exaggerated grain growth, because in the close-packing direction (0001, c-plane) the growth rate of the hexagonal crystal is very high. In this case some grains can increase their length and form rod-like crystals, absorbing neighboring crystals [58]. Since it happened, dense packing of grains is disturbed and more pores in microstructure are created. The evidence of rod-like grains’ existence can be seen in Figure 3 and Figure 4.

**Figure 6:**
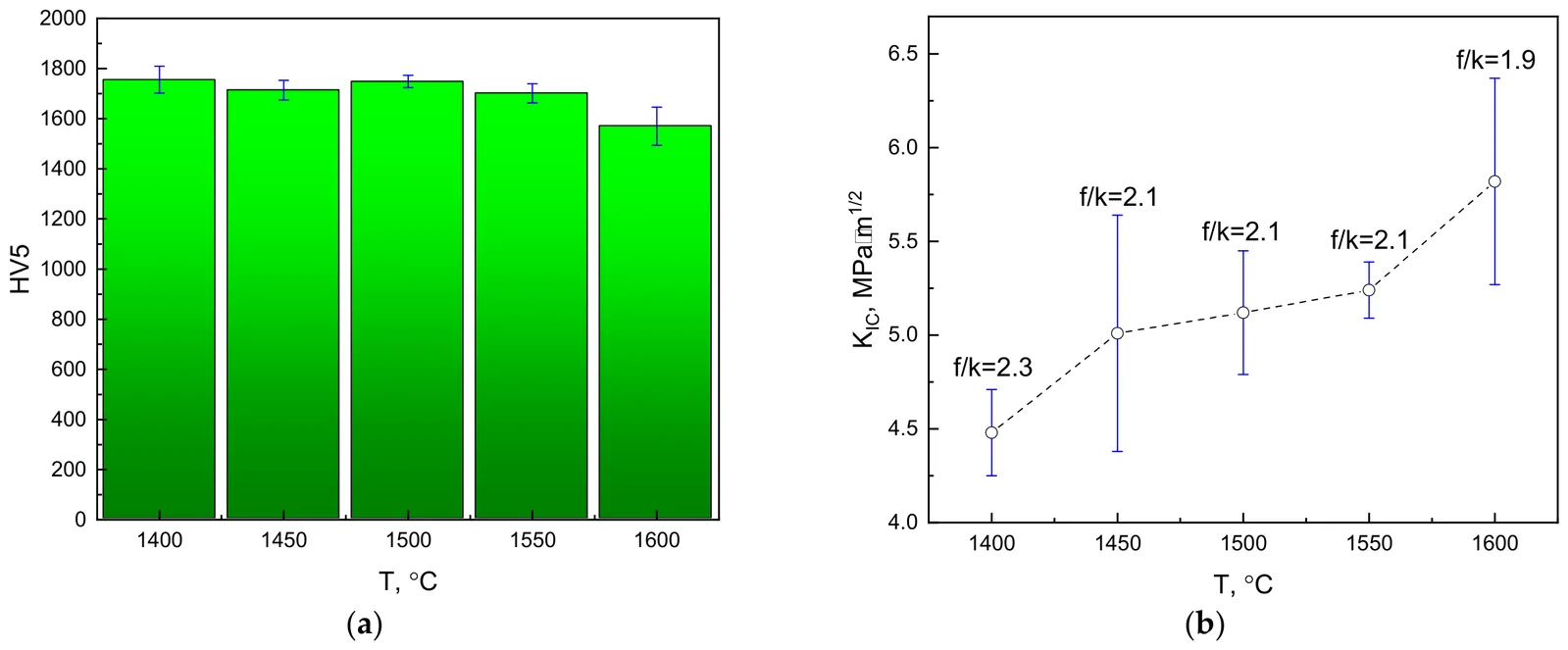
Dependence of (**a**) Vickers microhardness and (**b**) fracture toughness K_IC_ of MZTA samples on sintering temperature.

The variation in the mechanical properties of MZTA ceramics with increasing sintering temperature is primarily governed by microstructural evolution. The microhardness of the samples (Figure 6a) varies relatively little, ranging from 1570 to 1755 HV5. The lowest value of 1570 ± 76 HV5 was recorded for the MZTA sample sintered at 1600 °C. This reduction in microhardness is accompanied by pronounced grain growth of both Al_2_O_3_ and ZrO_2_. Grain coarsening and the associated decrease in grain-boundary area per unit volume may reduce the resistance to localized deformation during indentation, providing one possible explanation for the observed decrease in hardness. Moreover, according to the Hall–Petch relationship, finer grains improve hardness and strength by impeding dislocation motion. Excessive sintering temperatures promote grain coarsening, which degrades mechanical properties [45,59].

**Figure 7:**
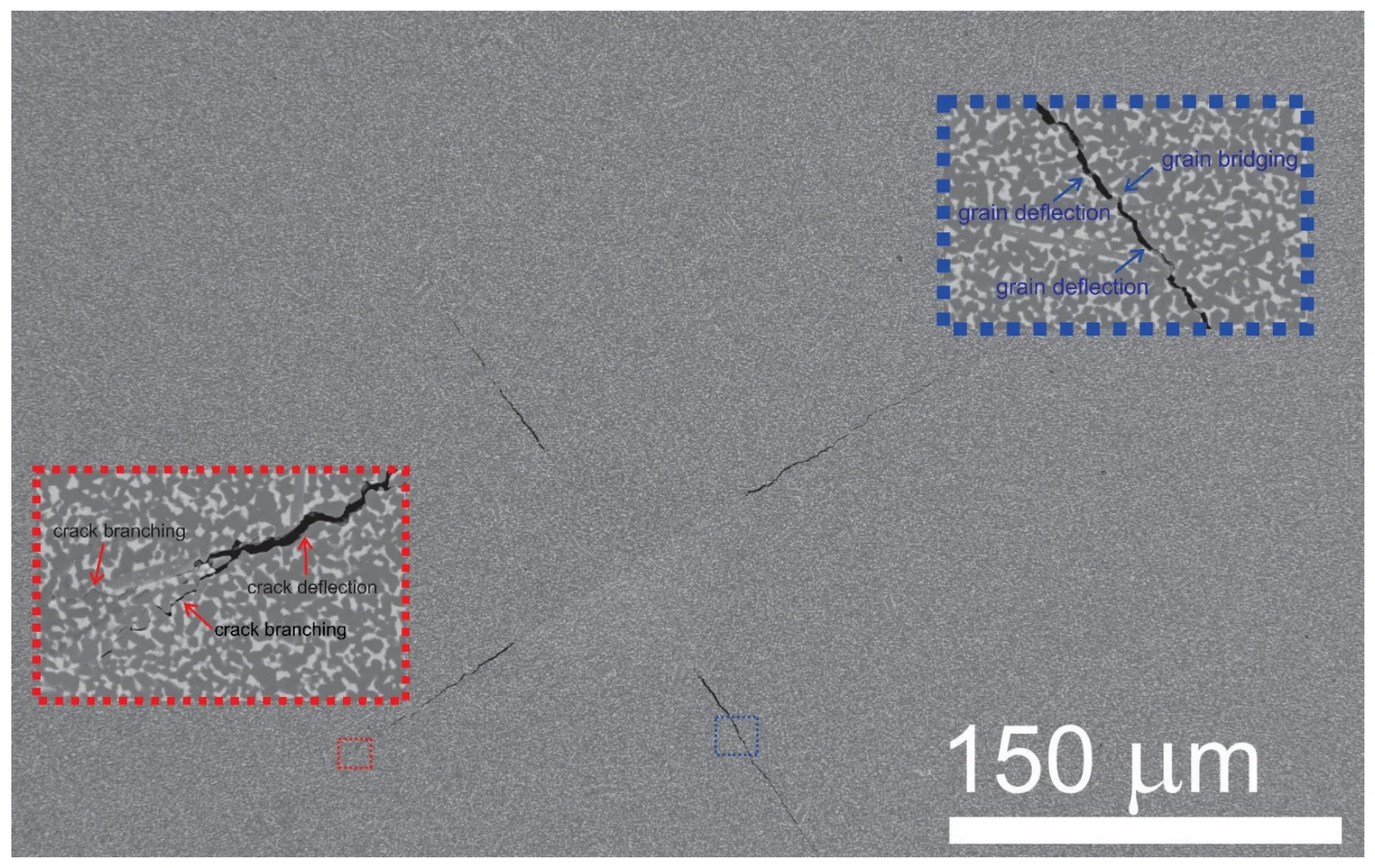
SEM image of the surface of the MZTA sample sintered at 1500 °C after Vickers indentation at HV5.

In contrast, the fracture toughness (K_IC_) values (Figure 6b) gradually increase with sintering temperature, reaching a maximum at 1600 °C. This trend may be attributed to the combined contribution of several toughening mechanisms, whose effectiveness changes as the microstructure evolves at higher sintering temperatures. The first mechanism is associated with changes in the stability of the t-ZrO_2_ phase as the average grain size increases. It is known that when the t-ZrO_2_ grain size exceeds approximately 0.4–0.5 µm, the energy barrier for the stress-induced t → m phase transformation decreases, facilitating the transformation toughening mechanism [60]. The volume expansion connected to the t → m phase transformation generates compressive stresses near the crack tip, thereby hindering further crack propagation. A second contribution to toughening arises from crack deflection and branching, as well as grain bridging. As shown in Figure 7, the crack propagation path is highly tortuous, with frequent changes in direction and secondary branches forming in some regions. Interactions of the propagating crack with grain and interphase boundaries, together with the increase in average grain size at higher sintering temperatures, elongate the effective crack propagation path and thereby promote additional energy dissipation. Grain bridging, in turn, produces crack-closing stresses behind the crack tip, further contributing to the increase in fracture toughness.

**Figure 8:**
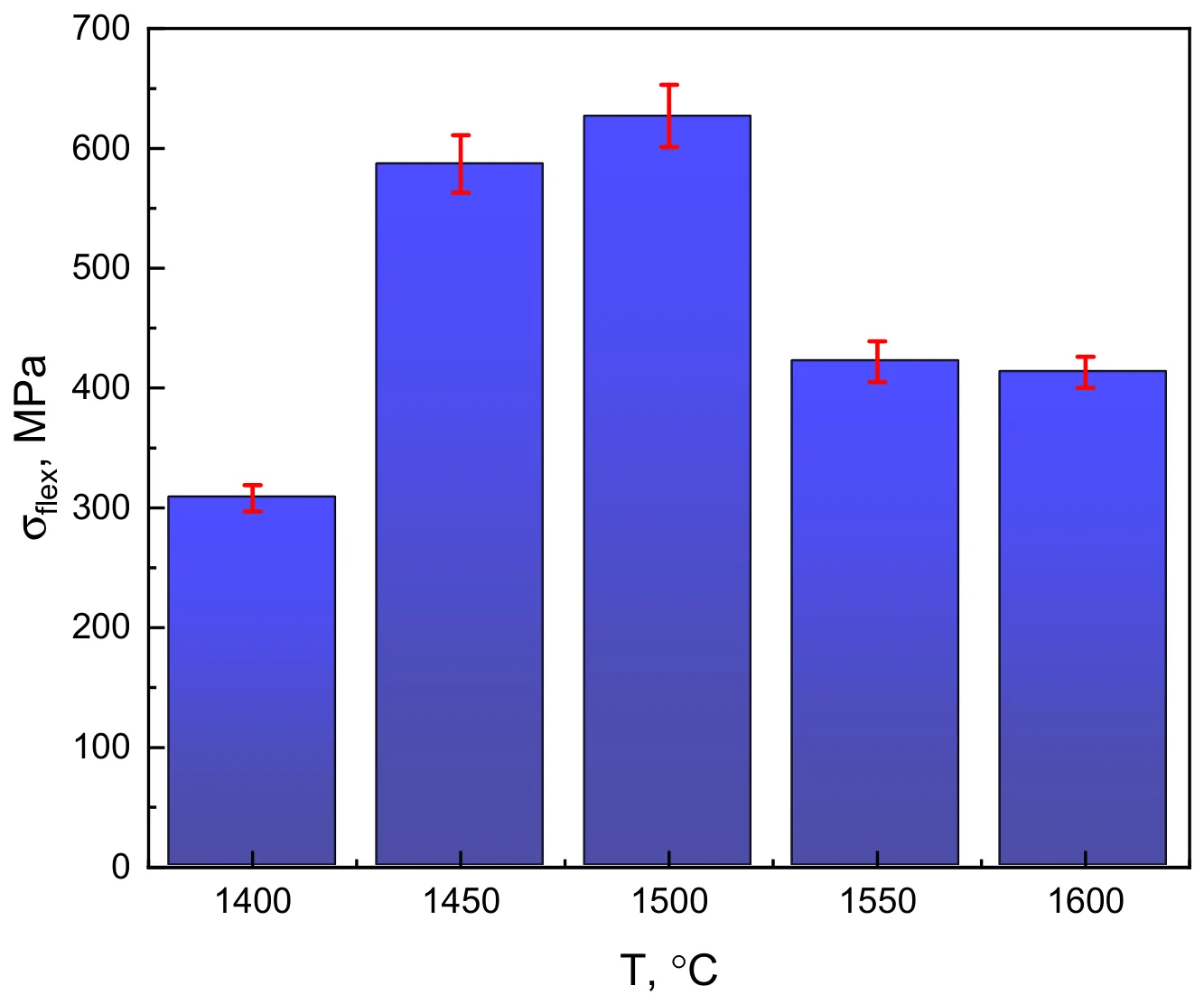
Dependence of the flexural strength σ_flex_ of MZTA samples on sintering temperature.

The flexural strength σ_flex_ exhibits a non-monotonic dependence on sintering temperature (Figure 8). Increasing the temperature from 1400 to 1500 °C results in a gradual improvement in strength, with the maximum value observed for the MZTA-1500 sample. Further heating to 1550–1600 °C is accompanied by a decline in σ_flex_. The initial improvement in flexural strength may be attributed to the development of a denser and more homogeneous microstructure during sintering. A reduction in the number and size of critical defects decreases the probability of crack initiation under flexural loading and consequently enhances the strength. The decline in σ_flex_ above 1500 °C may be associated with a transition from predominantly densification-driven microstructural evolution to intensive grain growth.

According to the SEM observations, increasing the sintering temperature is accompanied by substantial growth of both Al_2_O_3_ and Zr_0.90_(Y, Ce, Mg, Ca)_0.10_O_2_ grains, as well as the appearance of regions enriched in Zr_0.90_(Y, Ce, Mg, Ca)_0.10_O_2_. Such grain coarsening can contribute to a reduction in flexural strength and hardness, consistent with the Hall–Petch relationship, as reflected in the observed dependence of σflex on sintering temperature [61]. Greater microstructural heterogeneity may result in larger potential defects and more pronounced local stress concentrators, which can act as crack-initiation sites. According to brittle fracture mechanics, larger critical flaws reduce the stress required to initiate fracture.

**Figure 9:**
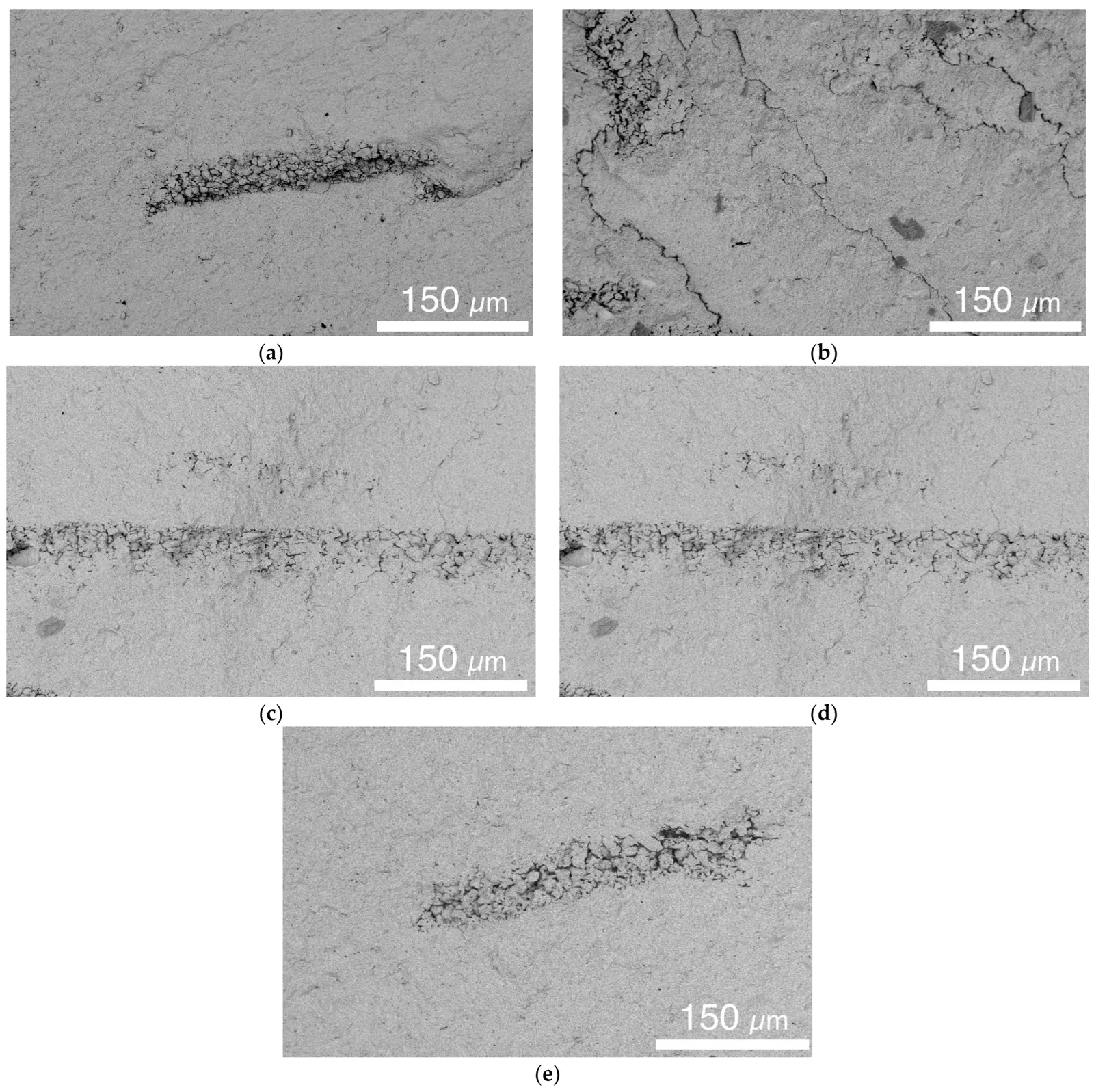
SEM images of the fracture surfaces of MZTA ceramics after flexural testing at different sintering temperatures: (**a**) 1400 °C, (**b**) 1450 °C, (**c**) 1500 °C, (**d**) 1550 °C, and (**e**) 1600 °C.

Meanwhile, examination of the fracture surfaces of MZTA ceramics after flexural testing (Figure 9) revealed regions of high residual porosity that were non-uniformly distributed throughout the ceramic volume. These porous regions demonstrated predominantly elongated and clustered morphologies, with characteristic dimensions ranging from approximately 10 to 100 µm. Such regions may act as local stress concentrators, promoting crack initiation and subsequent propagation under flexural loading.

## 4. Conclusions

In this study, the effect of sintering temperature in the range of 1400–1600 °C on the phase composition, microstructure, and mechanical properties of MZTA ceramics based on Al_2_O_3_ and multidoped Zr_0.90_(Y, Ce, Mg, Ca)_0.10_O_2_ was investigated. X-ray diffraction analysis showed that variations in sintering temperature had no significant effect on the phase composition of the MZTA ceramics. All samples consisted predominantly of α-Al_2_O_3_ and t-ZrO_2_, with only a minor fraction of m-ZrO_2_.

As the sintering temperature increased from 1400 to 1600 °C, the average grain size of Al_2_O_3_ increased from 0.39 to 1.32 µm, while that of ZrO_2_ grew from 0.28 to 0.92 µm. This grain coarsening was accompanied by a reduction in surface porosity. A characteristic feature of all the investigated samples was the presence of elongated, rod-like Al_2_O_3_ grains. Local EDS analysis revealed the presence of Y, Ce, Mg, and Ca in regions associated with these rod-like grains, suggesting possible redistribution of the dopants during sintering.

The microhardness of the synthesized MZTA samples was only weakly affected by sintering temperature and remained within the range of 1570–1755 HV5. In contrast, the fracture toughness K_IC_ gradually increased with temperature, reaching its maximum value at 1600 °C. The flexural strength σ_flex_ exhibited a non-monotonic dependence, with the highest value obtained at 1500 °C. This behavior is mainly governed by the competition between densification and grain growth. At elevated sintering temperatures, intensive grain growth promotes pore entrapment at grain boundaries and within the grain interiors, resulting in a reduction in the bulk density of the samples.

Thus, increasing the sintering temperature of MZTA ceramics has different effects on fracture toughness and flexural strength. A sintering temperature of 1500 °C provides the highest flexural strength, whereas further microstructural coarsening at 1600 °C enhances the contribution of transformation and microstructural toughening mechanisms, resulting in the highest fracture toughness.

## Figures and Tables

**Table 1:** Phase composition of MZTA ceramics sintered at different temperatures.

T, °C	α-Al_2_O_3_, wt. %	t-ZrO_2_, wt. %	m-ZrO_2_, wt. %	χ^2^
1400	62.7	37.3	–	2.008
1450	62.8	36.4	0.8	1.916
1500	62.6	34.6	2.8	2.207
1550	64.2	35.8	–	1.619
1600	65.8	33.5	0.7	1.436

## Data Availability

The original contributions presented in this study are included in the article. Further inquiries can be directed to the corresponding author.
